# The Enferplex Bovine TB antibody test shows a significant degree of positivity in both restricted and officially tuberculosis free herds in a national study of bulk tank milk samples from dairy herds in Great Britain

**DOI:** 10.3389/fimmu.2026.1779235

**Published:** 2026-10-28

**Authors:** Alastair Hayton, Phivos Sofokleous, Rachel Hayton, Eamon Watson, Lorraine McGilp, Emma O’Neil, Amanda O’Brien, Keith Cutler, Mike Coffey, Georgios Banos, Gordon D. Harkiss

**Affiliations:** 1 Synergy Farm Health Ltd, Dorset, United Kingdom; 2 SRUC Department of Animal Breeding and Genomics, Scotland’s Rural College (SRUC), Edinburgh, United Kingdom; 3 National Milk Recording, Glasgow, United Kingdom; 4 Enfer Scientific, County Kildare, Ireland; 5 MV Diagnostics Ltd, Roslin Innovation Centre, University of Edinburgh, Easter Bush Campus, Edinburgh, United Kingdom

**Keywords:** antibody, bovine tuberculosis, bulk milk, cattle, dairy, surveillance

## Abstract

**Background:**

Herd level bovine tuberculosis (bTB) surveillance worldwide is based on tuberculin testing (TT). The World Organisation for Animal Health has approved Enferplex Bovine TB antibody test (EP) for diagnosing bTB using bulk tank milk (BTM) samples and the use of this methodology provides the potential for a more resource efficient system of herd surveillance for bTB than is the case with TT. We aimed to assess whether there would be any benefit of routine BTM testing in Great Britain (GB) with the EP to the surveillance and control of bTB.

**Methods:**

We enrolled 4,830 dairy herds. Three sets of BTM samples were collected at three-month intervals from each herd without knowledge of their bTB status and analysed independently. A smaller cohort of 73 herds, that provided monthly samples over six months, was independently recruited. To provide a high confidence in the positive results, the risk of false positive results was minimised via minor adjustments to the test algorithm. To examine the impact on surveillance we assessed the prevalence and incidence of EP positive herds by the bTB risk area they were located and their bTB status based on Government surveillance data.

**Results:**

Twenty-five percent of herds tested positive at least once. In herds under restriction throughout the study, and whose tuberculin reactor animals had been removed (n=240), the test detected 40.4% (95% CI: 34.2-46.9) as positive. Monthly BTM sampling significantly increased the percentage of positive herds over quarterly sampling from 45% (95% CI: 26.9-65.4) to 64% (95% CI: 42.9-80.4) (P< 0.001). In herds designated as Officially Tuberculosis Free (OTF) throughout the study, 19.4% (95% CI: 18.1-20.7) tested positive.

**Conclusion:**

EP BTM testing registers substantial proportions of restricted and OTF herds as positive. EP adoption in surveillance programmes offers the potential for an overt improvement in bTB detection and thereby control.

## Introduction

Bovine tuberculosis (bTB) is a chronic bacterial disease of animals and humans caused by *Mycobacterium bovis* (*M. bovis*) and has a worldwide distribution (1). Of sixty-one countries that reported their bTB status through the World Animal Health Information System (WAHIS) from January 2017 to June 2018, 97.6% reported that tuberculosis was present within their livestock (2). In some low- and middle-income countries, especially where consumption of unprocessed milk is a common practice, it is a significant public health issue due to its zoonotic potential, causing an estimated 10% of human tuberculosis cases (3–5).

*M. bovis* is thought to be transmitted mainly via inhalation of aerosols produced by infected animals (1). Following uptake by alveolar macrophages, the bacteria gain access to lung tissue where dendritic cells (DC) transport the infection to local draining lymph nodes. Following antigen presentation by the DCs, cell-mediated immune responses (CMI) are induced which are mainly responsible for controlling the infection. This manifests itself as a delayed-type hypersensitivity reaction mediated by type 1T helper cell lymphocytes (TH1). These cells release pro-inflammatory cytokines such as interferon gamma (IFNg) and tumour necrosis factor and engage in a variety of cell-to cell signalling events aimed at controlling the infection. If the bacteria are not eliminated these CMI responses are thought to induce inflammation and lesions at the site of infection. The production of antibodies requires activation of a T helper cell subset known as T follicular cells (Tfl). This occurs when *M. bovis* antigens are released by infected macrophages and engage and activate B lymphocytes (6). These cells go on to produce antibodies only after these have been activated by Tfl cells. The timing and appearance of CMI and antibody responses is thus dependent on the availability of intra-macrophage and extra-cellular antigen respectively, but it can be as early as 3–4 weeks post-infection (7, 8). If CMI responses are controlling the availability of antigen, then antibody responses will be low. If they are not controlling antigen production antibody responses will be high. However, the host-pathogen interaction is dynamic, and therefore fluctuations in both TH1 and Tfl/B cell responses will occur. In both human and bovine TB, individuals vary in how well they control the infection leading to different timescales and patterns of progression. This will be reflected in presence and magnitude of the CMI and antibody responses detected. In addition, CMI responses have been shown to decline over time in individuals that do not appear to be controlling the disease, resulting in little or no CMI response and high antibody responses. The reasons for this are not clear but immunosuppressive and regulatory processes are likely to be involved (9).

The tuberculin skin test (TT) is the standard method for detecting tuberculosis in live cattle to determine population and individual animal freedom of infection as well as infection prevalence (5). This system of surveillance is usually supported by routine abattoir surveillance for bTB lesioned animals at commercial slaughter. Where a herd is disclosed as being under suspicion of, or confirmed for, the presence of bTB by this surveillance system, then the herd’s Officially Tuberculosis Free (OTF) status is removed, and the herd is placed under statutory bTB restrictions. In GB, consisting of England, Scotland and Wales), ancillary blood tests, in particular the IFNg test, are also employed on occasions by the Animal and Plant Health Agency (APHA) on behalf of the Competent Authorities (CA) to supplement the TT in bTB infected herds.

The WOAH lists three different forms of the TT: the comparative tuberculin skin test (CCT), which uses partially purified derivative of bovine (PPD-B) and avian (PPD-A) tuberculin, the single intradermal cervical tuberculin (SICT) test, and the caudal fold tuberculin test (CFT). All three tests have different diagnostic performance characteristics (10, 11). Whichever TT is used to establish herd freedom, the inherent requirements in the performance and reading of the test by a suitably qualified person, normally a veterinary surgeon, results in significant cost and resource use for both the farmer and the State. It has been estimated that approximately 80% of bTB control costs are due to performing the TT by veterinarians and that only 25% of affected countries were applying the relevant bTB control measures (12). WOAH report that appropriate control strategies are lacking in most low- and middle-income countries despite evidence of a high prevalence of bTB due to high cost and minimal or no appropriate resources (13).

The control of zoonotic tuberculosis is considered a priority area in aiming to end the global human TB epidemic with the agreed strategy, the *Roadmap for Zoonotic Tuberculosis*, set out by the WOAH, the Food and Agriculture Organisation (FAO) and International Union against Tuberculosis and Lung disease (14). Both the WOAH and the International Research Consortium on Animal Health (STAR-DIAZ) have stated that one of their main research priorities was to develop innovative diagnostic tests for both ante-mortem and post-mortem surveillance in various regions, including low- and middle-income countries.

While in general the specificity of TT in cattle, especially the CCT, is high (15) making it a useful tool for herd surveillance of bTB, there is considerable debate on the sensitivity of the TT tests at the individual and herd level. At the individual animal level published sensitivities range from 40.0% to 100%, 63.2% to 93.0% and 80.2 to 100% for the CCT, CFT and SICT tests respectively (11, 16), though Bayesian analysis of the SICT yielded a range from 53.0 to 69.4% (17). At the herd-level the sensitivity of the CCT was reported to be 61.3% (18). The low to moderate sensitivity of the CCT has been highlighted in several reports along with the consequent potential for this to result in recurrent bTB breakdowns (19, 20) and was identified as a key weakness of the control and eradication of bTB in the UK in the Bovine TB strategy review for England (21). The UK Government committed in their response to this report to improving diagnostic testing to identify bTB infected animals more effectively (22).

Consistent with the above aims, antibody tests have been developed to detect bTB over the last few years (23–25). There remains however a persistent view within the regulatory and the policy field that detection of *M. bovis* infected animals via the detection of antibody responses is of significantly less value than via use of CMI responses (10). This viewpoint stems from the conception that CMI responses predominate post infection (11), and that antibody production only occurs in the later stages of infection as the animal progresses to disease, albeit it is accepted that use of antibody tests does allow for detection of animals at this later stage of infection that are no longer producing a detectable CMI response (10). However, there has been increasing evidence in recent years, from both human and animal research, that antibody responses are an integral part of the immune response to Mycobacterium tuberculosis complex organisms from the point of infection (26–28) and that detectable antibody responses can be demonstrated starting at 3–4 weeks after infection in cattle (29–31). Further, it is well established that a combination of CMI and humoral based tests using anamnestic samples will significantly improve the sensitivity of detection of *M. bovis* infected animals (32–34) with studies demonstrating that injecting purified protein derivative of bovine tuberculin (PPDb), as part of the skin test, results in antibody levels rising to maximum around 3 weeks post injection. This boosting effect is dependent on the antigens used in any diagnostic test being present in PPDb. The degree of boosting for each antigen will be dependent on the production and level of specific B memory cells generated for each antigen. This will result in variable boosting effects being observed with different antigens. The boosting effect tends to tail off after about 4 weeks, though some studies show antibody responses being elevated for longer periods (8).

Two humoral based tests have been approved by WOAH for detection of bovines infected with *M. bovis*: the IDEXX antibody test and the EP. The IDEXX antibody test uses a mixture of MPB70 and MPB83 *M. bovis* antigens, while the EP test is a multiplex bTB antibody ELISA that uses an array of 11 separate *M. bovis* antigens, including MPB70 and MPB83 as well as PPDb. Comparisons between the two tests have shown that the EP test has higher sensitivity than the IDEXX test using anamnestic samples (35, 36). The EP test has demonstrated high sensitivity and specificity (37–39) and received WOAH approval for individual animal serum in 2019 and individual milk samples in 2023 and as a screening test to identify herds with *M. bovis* infection using BTM in May 2023.

It is important to emphasise that both CMI and humoral assays detect the host immune response to *M. bovis* infection rather than the pathogen itself. Consequently, these tests provide indirect evidence of infection and should be distinguished from direct pathogen detection methods, such as culture or molecular identification of *M. bovis*. A positive result from a CMI or humoral test therefore does not, in isolation, constitute direct confirmation of the presence of *M. bovis*. However, where such assays demonstrate high specificity, whether at the individual animal or herd level, a positive result is associated with a correspondingly high probability that *M. bovis* infection is present. This principle underpins the application of statutory bTB control measures by CA following the disclosure of positive test results from these assays. Evidence for clearance of infection in cattle is lacking and sparse for humans. While clearance may occur leaving residual CCT, IFNg and antibody responses, there is no current way of discriminating infection from residual immune responses.

The use of BTM testing as a routine, convenient surveillance tool for infectious disease in dairy herds is well established veterinary practice (40, 41) allowing not just a binary result for the presence or absence of disease but also, via its longitudinal use, an insight into the dynamics of the level of infection in the herd. As sample collection does not cause any disruption to individual farms or require significant technical human resources, bTB surveillance using BTM and the EP is both practical and feasible. Although there are very few publications examining antibodies in BTM in herds with bTB (42, 43), this approach could provide an inexpensive, rapid, regular, convenient and objective methodology, opening opportunities to introduce or enhance bTB surveillance in affected countries. To understand better the EP test’s potential field application, we conducted a large-scale longitudinal study on approximately two-thirds of dairy cattle farms in GB. The overall aim of this study was to assess the prevalence and incidence of EP positive bTB restricted and OTF herds by the bTB risk area in which they were located via their official bTB status based on Government surveillance data. To ensure maximum confidence in the study results, we first evaluated and minimised the false positivity rate (FPR) of the EP test bTB-free GB herds. The specific aims of the study were to 1) optimise the FPR of the test for GB samples, 2) determine the proportion of herds designated OTF at the time of BTM sampling that would test positive, 3) evaluate its ability to detect potential infection in herds under restriction, and 4) ascertain whether serial EP testing of BTM samples increased the proportion of test-positive herds. ARRIVE 10 guidelines were applied to the study experiments.

## Materials and methods

### Study design

Since the main objective of the study was to evaluate the potential benefit, if any, that BTM testing with the EP could bring to the surveillance and thereby control of bTB in GB, we aimed to enrol a significant proportion of dairy farms from across GB to ensure that bTB epidemiology would be captured in the dataset. All farms were provided with a document that summarised the proposed study and its aims and the regulatory implications. Two sets of herds were recruited: herds that underwent quarterly testing and a smaller independent set for monthly testing. Permissions to perform testing of BTM samples for antibodies to *M. bovis* antigens by EP were given by the relevant CA, namely, the Department for the Environment, Food and Rural Affairs (Defra) in England, the Scottish Government, and the Welsh Government. Country specific data were excluded from these permissions and consequently all data from the individual farms participating in the study were analysed according to the bTB risk area that the farm was situated in. Individual farmer permission for the quarterly tested farms was gained via an opt-out communication from their respective milk processor, while for the monthly tested herds via an opt-in communication with their private veterinary surgeon.

*Quarterly testing*. A sample of dairy herds (n=5,050) for quarterly testing were recruited with the aid of GB milk processors. BTM samples were collected by NMR Ltd three times at approximately three-monthly intervals starting in February, June, and October 2023 respectively from BTM samples submitted as part of the routine payment testing scheme for processors. The mean interval between the first and second round of sampling was 89 days and between the second and third round of sampling 99 days (**Figure 1**). Specifically, the BTM samples were collected from farms and delivered to NML Wolverhampton which is the laboratory used for all the routine payment testing of bulk milks for all dairy farms in GB. A schedule of sample collection for each enrolled farm was set up by NML Ltd, and the required samples were removed from the daily payment testing sample collection and these were then forwarded on a daily basis, via refrigerated transfer, to NML Hillington, Glasgow, UK. On arrival at NML Hillington, the samples were centrifuged, defatted and aliquoted into master sample blocks then stored at -20C until they were thawed and tested. The monthly samples were also dispatched to and tested at the NML at Hillington. Due to a technical problem, the first round of quarterly sampling was repeat tested, by the same NML Hillington laboratory team that performed the testing in Glasgow, at the Enfer Scientific Laboratory in Naas, Ireland.

**Figure 1:**
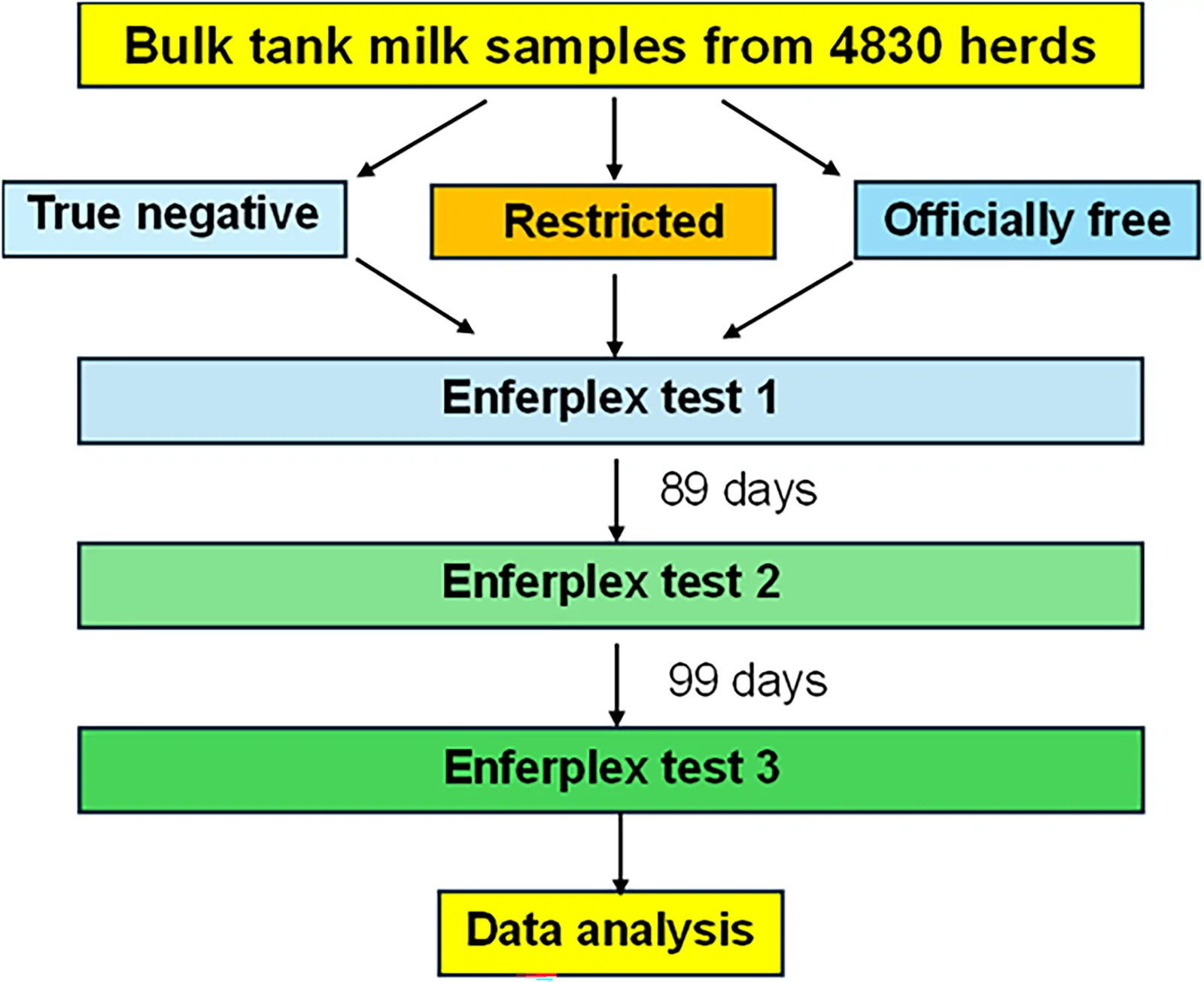
Flowchart showing study design. Samples were collected in three rounds approximately three months apart. True negative, bTB-free herds; Restricted, bTB restricted herds; Officially free, officially tuberculosis free herds over nine months.

The reason for retesting round 1 samples in the Enfer laboratory was down to laboratory logistics, commitments and costs. The Enferplex test reader and nine channel shaking incubator had been returned to the Enfer laboratory for use in other projects and it was deemed easier and cheaper for NMR staff to run the test in the Enfer laboratory than for Enfer to set up and optimise the test equipment again in the NMR laboratory. The NMR staff had been trained and were proficient in handling the equipment and performing the assay. The positive control values registered for each antigen in the repeat testing of round 1 samples in the Enfer laboratory were assessed in a random selection of plates of the run. The relative light unit (RLU) values registered for the duplicate positive controls showed coefficients of variation between 0.2 – 7.5% in these plates and the positive control values were within the test specification indicating that the repeat testing results obtained by the NMR staff at the Enfer laboratory were reliable. Similarly, the positive control values for Rounds 2 and 3 obtained in the NMR Hillington laboratory were also within the test specification. These results were consistent with the WOAH validation of the test using bulk milk samples which demonstrated a high level of reproducibility between three independent participating laboratories (39).

The results from these three rounds of testing were sent to Scotland’s Rural College (SRUC) where they were combined with bTB national surveillance data from the APHA. After matching, the total study dataset comprised 4,830 herds, representing approximately 64.8% of the 7,450 dairy holdings in GB (44). The design was blinded at several points: recruitment of farmers was voluntary, the processors and NMR were unaware of the bTB status of the herds, and the collation, analyses and reporting of results was performed independently by SRUC who hold a license to access the APHA bTB data. The results were reported by SRUC.

The bTB risk area analyses set out in this report have been produced by amalgamation of the following datasets: the high-risk area (HRA) category incorporated farms from the HRA in England and the East and West High TB regions of Wales. The edge area (EA) category combined farms from the EA of England and the intermediate Mid and North regions of Wales. The low-risk area (LRA) category combined the LRA of England and Low-TB area of Wales and all Scottish farms (as Scotland has had OTF country status within GB since 2009). Given these individual country risk areas are not entirely analogous in terms of their surveillance for bTB, incidence and prevalence of bTB and risk of loss of OTF status (and for the EA of England, not even within country, as this is itself subdivided into two regions), analysis of the study outcomes performed on this basis will inevitably be subject to some degree of error. The incidences and prevalences of bTB-infected herds, based on the current surveillance systems, are similar across the three amalgamated risk area categories used in this study and the relevant risk areas within individual country datasets (45). Therefore, combining these risk area datasets is unlikely to have significantly compromised the results or the conclusions drawn from them. Furthermore, this amalgamation has the advantage of providing a substantial increase in the power of many of the observations performed in the study due to the larger datasets that it encompasses.

*Monthly testing.* Monthly testing was performed on an independent set of 73 herds from the HRA (n=70) and EA (n=3). These herds were recruited by private veterinary surgeons, who were principally working at Synergy Farm Health Ltd, a veterinary practice based in the South-West of England, and therefore working with farms based in the HRA. Recruitment was focused on farms that were milk recording with NMR Ltd. There was no selection based on the bTB history of the farm. However, as the farms responded to newsletter requests or following discussion with their veterinary surgeons, they were more likely to be concerned about the risk of bTB. The group consisted of 51 OTF herds and 22 herds that were under restriction for part, or the whole period, of the study. Samples were taken every month for 6 months and tested for antibodies by the EP. The study design was similar to the quarterly testing (**Figure 1**) except that testing was performed monthly for 6 months. NMR were unaware of the bTB status of the farms supplying monthly samples.

### Sample size

Since the recruitment process was random and blinded, without any knowledge of bTB status, it was not possible to calculate minimum sample sizes for each study category. We adopted the pragmatic approach of recruiting as many herds as possible to obtain sample sizes large enough to allow assignment of statistical significance. We achieved recruitment of 65% of the GB dairy herds resulting in a total of the 4,830 herds for the study. The number of experimental units in each group was determined by data extraction from the APHA bTB database, which holds details of herd bTB status, duration, location, and history. This was done at the data analyses stage.

The average dairy herd size in England, Wales and Scotland in 2023 was 178, 181 and 220 respectively (44). The average milking herd size in the study based on milk recorded herds (n=1,724) was 194 +/- 2.4. Using the number of animals calved as a proxy, the average milking size of 4,830 herds in 2023 was 241 +/- 3.2. The milking herd size varied at each round of testing because it is dependent on the number of animals contributing to the BTM at any one point. This, in turn, will be largely a function of the calving pattern of individual herds.

### Experimental unit

The experimental unit was BTM collected from individual herds. BTM is the pool of all the saleable milk harvested since the last collection of such milk by the processor that the farmer wishes to supply to the processor. As results are reported at the herd level, herds with multiple bulk tanks, and therefore supplying more than one BTM sample at each individual sampling round, were considered EP positive at that round if one or more of the BTM samples tested positive. As the time point of tuberculin skin testing of participating herds in the study was not known at the point of sampling, the three main BTM sample sets (bTB free, OTF and restricted) included both samples taken outside the anamnestic window (n = 2,711; 56.1%) and within this window (n = 2,119; 43.9%).

### Experimental groups

The number of herds comprising the experimental groups are shown in **Table 1**. The main experimental groups that were tested quarterly were determined solely by the herds satisfying the criteria for each bTB status category in the bTB APHA database.

**Table 1:** Experimental groups analysed in the study.

Experimental groups^1^	Number of herds	Herd categorisation
Quarterly tested herds		
All herds^2^ - HRA - EA - LRA	4830 2514 753 1563	All herds participating in the study - High risk area. - Edge area. - Low risk area.
bTB free^3^ - All from LRA	365	BTM samples taken at any time during the study
OTF^4^ - HRA - -EA - LRA	3473 1401 552 1520	OTF throughout the whole study
bTB positive^5^ - HRA - EA - LRA	240 207 27 6	Under bTB restriction during the whole study
Monthly tested herds		
bTB positive^5^ - HRA - EA	22 (132 samples) 19 3	Monthly sampling of restricted herds for 6 months.
OTF^4^ - HRA - EA	51 (306 samples) 51 0	Monthly sampling of OTF herds for 6 months.

1 Herd status defined by tuberculin testing and/or slaughterhouse surveillance.

2 Herds that were true negative, OTF or restricted status.

3 bTB free – OTF herds from the LRA that contributed to the true negative dataset and were defined as being bTB free for at least 8 years and greater than 16 km from the nearest bTB breakdown in the last 12 months prior to, and during, the study period.

4 OTF– Officially tuberculosis free by statutory testing.

5 bTB positive – herds that were under restrictions.

In this study, bTB-free herds were defined as herds that had no bTB detected during at least the previous 8 years and were at least 16 km distant from the nearest bTB breakdown herd in the 12 months prior to the start of the study. The definition of a bTB-free herd, for the purposes of WOAH validation, required at least 8km distance from the nearest bTB breakdown. In our previous work we found that bTB-free BTM samples from the UK had a slightly higher false positive rate (FPR) than samples from OTF EU countries (39). Since bTB is endemic in certain regions of the UK, we anticipated a similar result in this study, which could compromise interpretation of EP positivity results found in restricted and OTF herds. To address this issue, we used an asymptotic regression methodology, set out by Goodchild and colleagues (15) to determine the FPR of the CCT test, to determine the optimal distance criterion for FPR determination in GB samples. We then re-assessed the individual antigen cut-offs for a GB setting to meet a target of <1% FPR.

OTF herds were defined as having passed their most recent statutory CCT testing, which takes place at different frequencies in each country and risk area of GB (45). Herds designated as bTB positive (or bTB breakdown herds) refer to herds that had failed a CCT test and/or had animals with lesions at post-mortem and were placed under animal movement restrictions. APHA epidemiological analysis of bTB data from England showed that 9% of incidents that resulted in the loss of OTF status in 2023 were due to abattoir surveillance rather than via on-farm CCT testing of herds or individual animals (46). In this study, 89% of the ‘bTB positive’ category was from herds that had lost their bTB status as a result of the CCT test and 11.0% because of bTB lesions being disclosed at slaughter. The monthly testing experimental groups are shown in **Table 1**. The same definitions for OTF and restricted herds apply as for the quarterly tested groups.

In restricted herds, where there is confirmation of *M. bovis* infection by APHA, due to a positive culture result, then these herds are classified as OTF-W (Officially Tuberculosis Free – Withdrawn). Herds under restriction for the suspicion of the presence of bTB, where that suspicion is not confirmed, or is still pending confirmation, are classified as OTF-S (Officially Tuberculosis Free – Suspended). However, analysis of the EP results in relation to this confirmatory data could not be performed as the APHA bTB dataset did not provide sufficient clarity on the herd’s OTF-S/OTF-W status for it to be certain as to an individual restricted herd’s status in relation to these two classifications.

### Enferplex test and outcomes

The principles and methodology of the EP test have been detailed in previous publications (23, 37–39). Briefly, the test conforms to a standard indirect ELISA format but with two differences: instead of wells being coated with a single antigen, 11 individual antigens are printed onto the surface of each well in a small planar array; and the endpoint is chemiluminescence rather than colorimetric. The light intensity emitted from each antigen spot is detected by digital image analysis and is converted into RLU. Each antigen has its own cut-off to determine whether the antibody response to that antigen is positive or negative. A sample is determined to be positive based on a two-antigen rule where a minimum of two antigens give RLU values above their individual set thresholds. Responses to one or zero antigens are deemed to be negative. Where there was more than one BTM sample from a farm a herd will have a result for each bulk tank, and the herd was deemed positive if one or more of the BTM samples was positive using the two-antigen rule. The outcome measure was the presence or absence of antibodies in the BTM samples as detected using the EP. Two sets of cut-offs were used to determine a high sensitivity (Hse) setting and a high specificity (Hsp) setting respectively (37–39) with the Hse setting requiring a lower RLU cut-off for an individual antigen response to be determined as positive as compared to the Hsp setting.

### Statistical analyses

Herd data from NMR were in Excel workbooks and CSV files. Scripts were written in Python (Python Statistical package version 3.14 Statistics - Mathematical statistics functions - Python 3.14.3 documentation) to process the data received from NMR. Python scripts were also used to produce the figures in the manuscript using the Matplotlib package. The project database was then joined with data from other national databases stored in Microsoft SQL Server databases hosted at SRUC for analyses. Non-parametric descriptive associations were based on Microsoft Excel, and analyses included descriptive associations and Chi-square test comparisons in tabular data. Parametric analyses were conducted with the ASReml software: ASReml User Guide Release 4.1 Structural Specification, VSN International Ltd, Hemel Hempstead, HP1 1ES, UK.

The monthly testing dataset was used to assess the impact of EP sampling frequency on EP positivity by sequentially removing actual monthly tests. Thus, the proportion of EP positive herds determined from all 6 monthly tests was compared to EP positivity based on only 1, 2, 3, 4 or 5 tests. In each case, the latter was calculated as the average of all possible combinations. For example, EP positivity from 3 monthly tests was the average of tests 1-2-3 (i.e. removing months 4-5-6), 1-2-4, 1-2-5, 1-2-6, 1-3-4, 1-3-5, 1-3–6 etc. The same data analyses were conducted separately on OTF and restricted herds. Statistical significance threshold was set at P=0.05 in all data analyses.

## Results

### Optimisation of selection criteria to minimise EP false positive results in bTB free herds

When analysed by distance from the sample herd to a herd that was under bTB restrictions, the estimated FPR at the herd level progressively dropped from 4.5% at 0 km to 0.8% as the distance from a breakdown herd increased and reached an asymptotic maximum around 16 km (**Figure 2**). We therefore adjusted the distance criteria within the classification of a true negative herd used in the WOAH validation of a distance of no less than 10km away from a herd under bTB breakdown to a distance of no less than 16km away and reassessed the false positive rate in the herds that met this new definition of true negative herds. This analysis showed that the FPR was still just below our target FPR of less than 1%.

**Figure 2:**
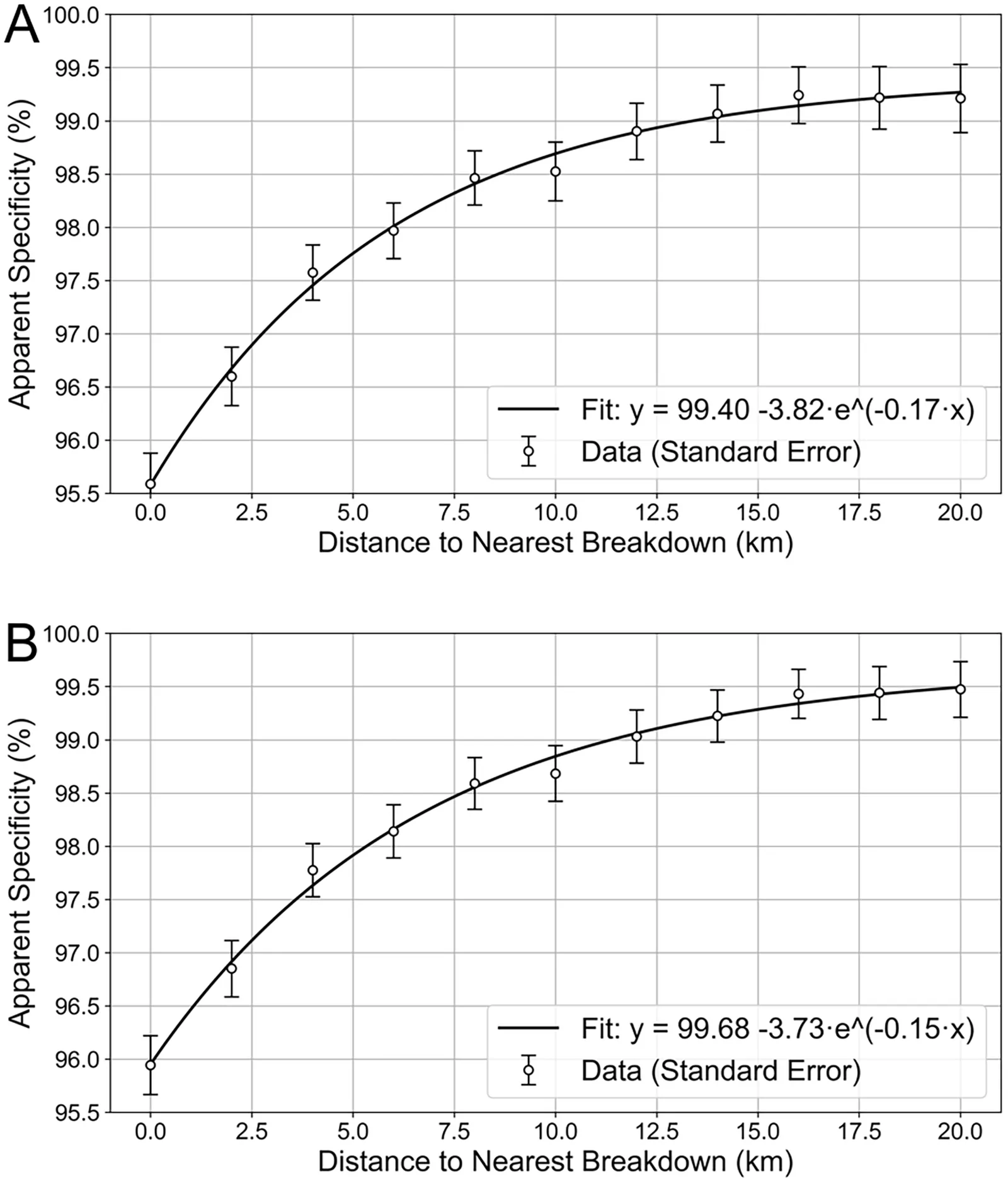
Asymptotic plots of the Enferplex Bovine TB antibody test positivity at the herd level in OTF herds for the Hse setting **(A)** and Hsp setting **(B)**. The results show the average EP positivity over three testing rounds against distance in km from nearest bTB breakdown in the last 12 months prior to start of the study with asymptotic regression fit of data. Bars indicate se.

Examination of the blanked RLU values in samples from herds that showed a positive result in these bTB free herds, indicated that some antigens had one or more RLU values close to the WOAH cut-off. The cut-offs of two of these antigens, with RLU values closest to the cut-off, were selected for modification. This minimised the effect of raising the cut-offs on the relative sensitivity of the test. It was found that raising the cut-offs of two antigens by factors of 1.27 and 1.15 provided results within our target FPR of less than 1%.

Prior to applying these new cut-offs to the full study dataset, we assessed the effect of these changes using the WOAH validation dataset to check that they would not have a profound negative effect on the sensitivity of the test. The WOAH validation gave a specificity of 99.8% in BTM samples using the original Hse cut-offs, and using the modified cut-offs identified in this current study, the specificity of the WOAH dataset would have been unchanged at 99.8%. The relative sensitivity of 77.7% observed in the WOAH dataset would be moderated to 76.1% using the modified cut-offs identified in the present study. We consequently felt confident that the changes to the cut-offs were appropriate and were adopted for all the subsequent analyses.

The results of assessing the FPR rate at the herd level in GB bTB free dairy herds at the Hse and Hsp settings of the test using these criteria are shown in **Table 2** for the Hse and Hsp settings respectively. A FPR of 0.8% was found overall. An FPR of 1.4% was found in Round 1 using the Hse setting compared to 0.3% and 0.6% in Rounds 2 and 3. No technical reasons for this were identified. To determine whether anamnestic samples were contributing to this result, the herds were segregated into anamnestic and non-anamnestic categories and analysed separately. The results obtained at the Hse and Hsp settings of the test are shown in **Table 3**. The proportions of anamnestic samples that were EP positive in Rounds 1, 2 and 3 were 10.5%, 0% and 0% respectively and 7.1% overall. The FPT in non-anamnestic samples was 0.9%, 0.3% and 0.6% for Rounds 1, 2 and 3 respectively and 0.6% overall. The results indicate that the higher FPR overall was mainly due to Round 1 samples where the FPR in anamnestic samples was 10.5% compared to 0.6% in non-anamnestic samples. Similar results were observed using the Hsp setting of the test.

**Table 2:** False positive rate of the EP using bulk tank milk samples from GB bTB-free dairy cattle herds^1^ at the Hse and Hsp setting of the test (16 km criteria).

Test setting^2^	Statistical variable^3^	Enferplex bTB antibody test positivity			
		Round 1	Round 2	Round 3	All 3 rounds
Hse	N	349	363	345	1057
	n	5	1	2	8
	%	1.4	0.3	0.6	0.8
	95% CI	0.6-3.3	0.0-1.6	0.2-2.1	0.4-1.5
Hsp	N	349	363	345	1057
	n	4	0	2	6
	%	1.1	0	0.6	0.6
	95% CI	0.4-2.9	0.0-1.1	0.2-2.1	0.3-1.2

1 Dairy herds with no history of bTB for at least 8 years and no bTB breakdown herds within 16 km in the last 12 months.

2 Hse, High sensitivity test setting; Hsp, High specificity test setting.

3 N, number of herds; n, number of herds positive, % - percent positive; n - 95% CI, 95% confidence interval.

**Table 3:** Diagnostic herd level specificity of the EP test at the Hse setting of the test (16 km criterion) using BTM samples from GB dairy cattle collected within and outside the anamnestic period of 5–30 days post tuberculin administration^1^.

Sample category^2^	Statistical variable^3^	Enferplex bTB antibody test positivity			
		Round 1	Round 2	Round 3	All 3 rounds
Hse: Non anamnestic	N	330	360	339	1029
	n	3	1	2	6
	%	0.9	0.3	0.6	0.6
	95% CI	0.3-2.6	0.05-1.6	0.16-2.1	0.7-3.4
	Dsp	99.10%	99.70%	99.40%	99.40%
Hse: Anamnestic	N	19	3	6	28
	n	2	0	0	2
	%	10.5	0	0	7.1
	95% CI	2.9-31.4	0.0-56.1	0.0-39.0	2.1-24.1
	Dsp	89.50%	100%	100%	92.90%
Hsp: Non anamnestic	N	330	360	339	1029
	n	2	0	2	4
	%	0.61	0	0.59	0.39
	95% CI	0.16-2.2	0.0-1.1	0.16-2.2	0.15-1.0
	Dsp	99.4	100	99.4	99.6
Hsp: Anamnestic	N	19	3	6	28
	n	2	0	0	2
	%	10.5	0	0	7.1
	95% CI	2.9-31.4	0.0-56.1	0.0-39.0	2.0-22.6
	Dsp	89.5	100	100	92.9

1 Hse, High sensitivity setting.

2 Dairy herds with no history of bTB for at least 8 years and no bTB breakdown herds within 16 Km in the last 12 months; ^2^Hse, anamnestic and non-anamnestic; Hsp – anamnestic and non-anamnestic.

3 N, number of herds; n, number of herds positive, % - percent positive; n - 95% CI, 95% confidence interval. Dsp - Diagnostic specificity.

### Enferplex bTB antibody positivity in all dairy herds by testing round

The results for EP positivity found in all dairy herds (n = 4,830) by testing round are shown in **Figure 3**. The results show that 18%, 6% and 2% of herds were EP positive at one, in two, or in all three rounds of testing respectively giving a total of 25% EP positivity across all 4,830 herds in the study.

**Figure 3:**
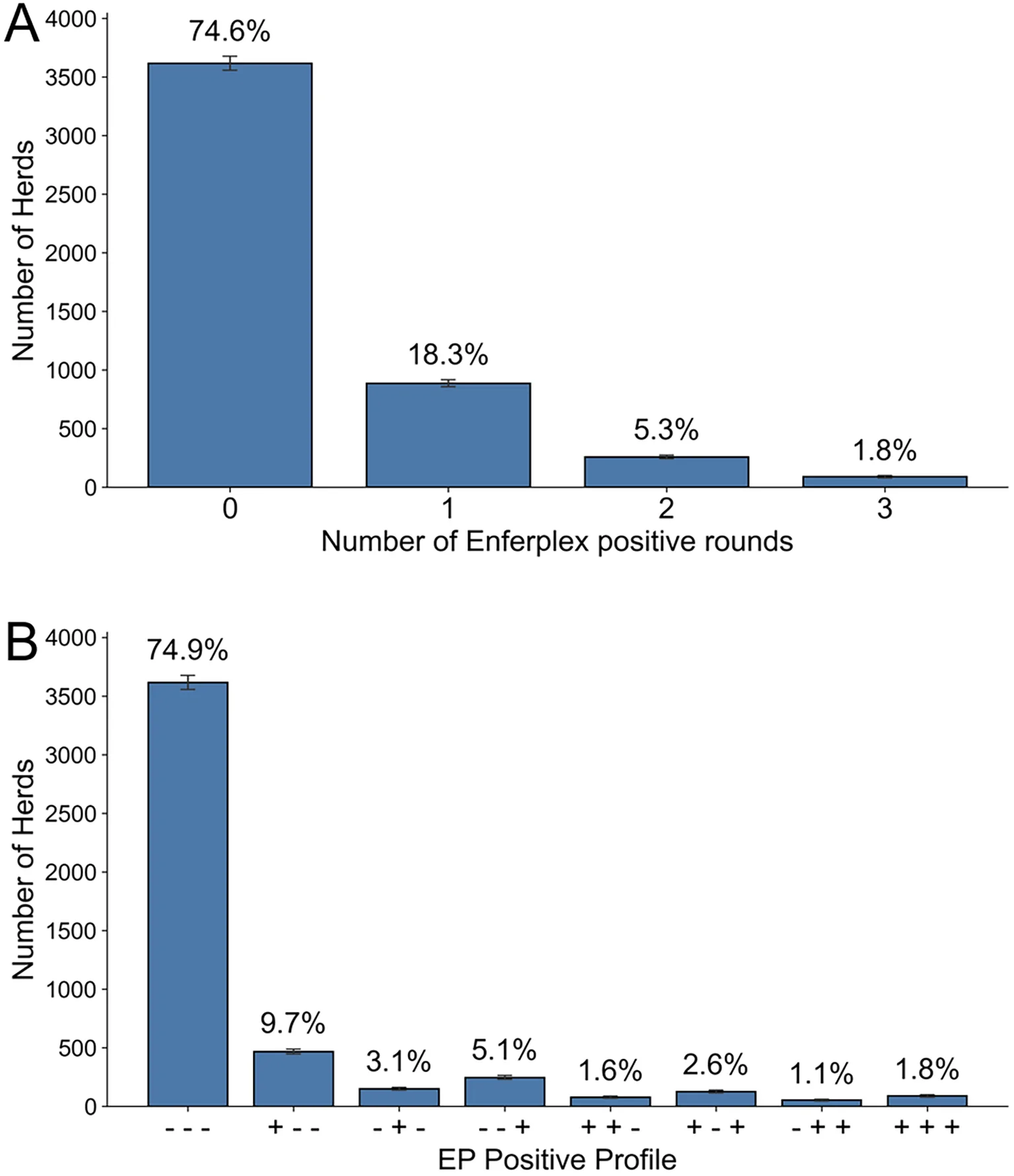
Number of herds EP positive by testing round. The EP negative herds are denoted 0, while the EP positive herds in each testing round are denoted 1, 2 and 3 **(A)**. Percentage of herds (n= 4,830) by their EP positivity profile per round of testing **(B)**. The EP result is denoted negative (–) or positive (+) for the three rounds of testing. The percentage EP positivity is shown above the bars (mean +/- se).

### Enferplex bTB antibody positivity in all dairy herds by bTB risk area

The results for EP positivity found in all dairy herds in the study are shown in **Table 4**. The results show that for all test rounds EP positivity was highest in the HRA (35.8%), followed by the EA (27.2%), then the LRA (7%). The EP positivity rate in HRA herds was significantly higher than in the EA, and LRA, as were the differences between the EA and the LRA (P<0.0001). The proportion of EP positive herds that came from the HRA, EA and LRA were 74.1%, 16.9%, and 9.0% respectively. Overall, 25.1% of herds tested were EP positive at least once and therefore EP demonstrated a very similar incidence of positive herds to that produced by the current statutory surveillance systems employed by the CA (28%). The relative risk of EP positivity was 9.2, 36.0 and 47.2 times higher in the LRA, EA and HRA compared to bTB-free herds (**Table 2**) respectively (P < 0.0001). The relative risk was 3.9 and 5.1 times higher in the EA and HRA compared to the LRA respectively (P < 0.001).

**Table 4:** Enferplex bTB antibody positivity from all study herds by GB bTB risk areas.

		Enferplex Bovine TB antibody test (Hse)^3^					
GB TB risk areas^1^	Statistical variable^2^	Round 1	Round 2	Round 3	EP positive at least once^4^	Risk relative to LRA	Risk relative to bTB free herds^5^
HRA	N	2211	2400	2197	2514	5.1	47.2
	n	563	296	390	899		
	%	25.5	12.3	17.8	35.8		
	CI	23.7-27.3	11.1-13.7	16.2-19.4	33.0-37.7		
EA	N	610	731	685	753	3.9	36
	N	122	59	90	205		
	%	20	8.1	13.1	27.2		
	CI	17.0-22.4	6.3-10.3	10.8-15.9	24.2-30.5		
LRA	N	1433	1532	1451	1563	1	9.2
	N	17	17	35	109		
	%	1.2	1.1	2.4	7		
	CI	0.7-1.9	0.7-1.8	1.7-3.3	5.8-8.3		
All	N	4254	4663	4333	4830	–	–
	N	762	372	515	1213		
	%	17.9	8	11.9	25.1		
	CI	16.8-19.1	7.2-8.8	11.0-12.9	23.9-26.4		

1 HRA - high risk area; EA, Edge area; LRA, Low risk area.

2 N, number of herds; n, number of herds Enferplex positive; %, percentage positive; 95% CI, 95% confidence interval.

3 Hse, High sensitivity setting.

4 Tested positive at least once during the study period.

5 Relative to EP positivity at least once in bTB free herds as shown in **Table 2**.

### Enferplex bTB antibody positivity in OTF dairy herds by round and risk area

The results for EP positivity found in OTF herds in the study are shown in **Table 5**. Of the herds designated as OTF throughout the study period, EP positivity was highest in the HRA (31.8%), followed by the EA (24.8%), then the LRA (5.9%). The EP positivity rate in HRA herds was significantly higher than the EA (P = 0.002) and LRA (P<0.0001). The differences between the EA and the LRA were also statistically significant (P<0.0001). The contribution to the EP positivity rate was 66.3% from the HRA, 20.4% from the EA and 13.2% from the LRA. Overall, 19.4% of dairy herds that were OTF throughout the study were EP positive. Across all herds 90% (95% CI: 87.5-92.1%) of OTF EP positive herds received a whole herd TT within the study period. When analysed by risk area in the HRA, EA and LRA, 96.6% (CI: 94.5 – 98.0%), 96.4% (95% CI: 91.7 – 98.4%) and 47.2% (95% CI: 37.2 – 57.5%) respectively received a whole herd TT within the study period. These tuberculin-tested herds, therefore, had the opportunity to have been disclosed via routine surveillance. The results also show that the relative risk was 4.2 and 5.4 times higher in the EA and HRA compared to the LRA respectively. The results show that the relative risk of EP positivity was 7.7, 32.8 and 42.1 times higher in the LRA, EA and HRA compared to bTB-free herds (**Table 2**) respectively (P < 0.0001). The latter was calculated by comparing the EP positivity rate observed in OTF herds (672/3,473) with that found in bTB free herds (8/1,057).

**Table 5:** Enferplex bTB antibody positivity at herd level in herds that were OTF^1^ throughout the study period by their GB bTB risk area location.

GB TB risk areas^2^	Statistical variable^3^	Enferplex bTB antibody test positivity (Hse)^4^					
		Round 1	Round 2	Round 3	EP positive at least once	Risk relative to LRA	Relative risk relative to bTB free herds^5^
HRA	N	1238	1344	1205	1401	5.4	42.1
	n	281	141	187	446		
	%	22.7	10.5	15.5	31.8		
	CI	20.5-25.1	9.0-12.2	13.6-17.7	29.4-34.3		
EA	N	445	535	499	552	4.2	32.8
	n	76	42	57	137		
	%	17.1	7.9	11.4	24.8		
	CI	13.9-20.9	5.8-10.6	8.8-14.6	21.4-28.6		
LRA	N	1391	1491	1415	1520	1.0	7.7
	n	65	10	29	89		
	%	4.7	0.7	2.1	5.9		
	CI	3.7-6.0	0.3-1.3	1.4-3.0	4.8-7.2		
All areas	N	3074	3370	3119	3473	–	–
	n	422	193	273	672		
	%	13.7	5.7	8.8	19.4		
	CI	12.6-15.0	5.0-6.6	7.8-9.8	18.1-20.7		

1 OTF, officially tuberculosis free as determined by statutory testing.

2 HRA - high risk area; EA, Edge area; LRA, Low risk area.

3 N, number of herds; n, number of herds Enferplex positive; % - percentage positive; 95% CI, 95% confidence interval.

4 Hse, High sensitivity setting.

5 Relative to EP positivity at least once in bTB free herds as shown in **Table 2**.

### Antibody positivity in herds restricted throughout the period of study by round and risk area

The EP positivity results obtained from herds that were restricted throughout the study period are shown in **Table 6**. The overall EP positivity was 40.4%. The results show that for the HRA EP positivity was 39.6% overall. The EP results from herds in the EA and LRA were not analysed statistically due to low numbers. The EP positivity rate was higher in Round 1 compared to Rounds 2 and 3 in all risk areas. The overall rate of positivity was higher in all the continuously restricted herds (40.4%; **Table 6**) than for all herds (25.1%; **Table 4**). The relative risk of restricted herds being EP positive was 50.5 times higher than if herds were bTB-free (**Table 2**; P<0.05). This figure was calculated by comparing the EP positivity rate in herds that were restricted throughout the study (97/240) with that observed in bTB free herds (8/1,057). For reference, in herds that were under restriction at the point of sampling but were not necessarily under restriction throughout the whole study period, the EP positivity was 477/1,151 (41.4%; 95% CI: 37.2-46.0).

**Table 6:** Enferplex positivity in herds that were restricted throughout the period of the study by their GB bTB risk area location.

UK TB risk areas^1^	Statistical variable^2^	Enferplex Bovine TB antibody test (Hse)^3^			
		Round 1	Round 2	Round 3	EP positive at least once^4^
HRA	N	173	200	180	207
	n	53	28	40	82
	%	30.6	14	22.2	39.6
	CI	24.0-38.2	9.7-19.8	16.5-29.1	33.0-46.7
EA	N	19	25	24	27
	n	9	1	6	11
	%	47.4	4	25	40.7
	CI	25.2-70.5	0.2-22.3	10.6-47.1	23.0-61.0
LRA	N	6	6	4	6
	n	4	1	1	4
	%	66.7	16.7	25	66.7
	CI	24.1-94.0	0.0-63.5	1.3-78.1	24.1-94.0
All areas	N	198	231	208	240
	n	66	30	47	97
	%	33.3	13	22.6	40.4
	CI	26.9-40.4	9.1-18.2	17.2-29.0	34.2-46.9

1 HRA, high risk area; EA, Edge area; LRA, Low risk area.

2 N, number of herds; n, number of herds Enferplex positive; %, percentage positive; 95% CI, 95% confidence interval.

3 Hse, High sensitivity setting.

4 Tested positive at least once during the study period.

### Comparison of EP positivity in OTF and restricted herds in relation to risk area

We used the association between official TB herd status and Enferplex test status using the chi square test for independence on the data in **Tables 5** and **6**. The overall result was Chi^2 (dof=1) = 217.8 (P < 0.001) indicating strong evidence of association. Stratifying the herds by risk area gives a 6 x 2 contingency table, with overall association remaining strong (Chi^2 (dof=5) = 495.3 (P < 0.001) with the largest contribution to chi-squared coming from the HRA restricted and LRA OTF categories.

### Enferplex bTB antibody test positivity in relation to the days prior to and post lifting of restrictions in newly defined OTF herds

The results of analysing EP positivity in relation to the days prior to and post lifting of restrictions in newly defined OTF herds are shown in **Figure 4**. During restriction, the EP positivity ranged between 15.4% to 35.4% with a mean +/- se of 24.6 +/- 2.4%. After regaining OTF status, the EP positivity ranged between 7.0% and 26.7%, with a mean +/- se of 14.4 +/- 2.1%. The difference in EP positivity between the restricted and OTF periods was statistically significant using Chi-square analysis (P < 0.0001). The EP positivity profile varied over time in both the restricted and OTF periods. The results clearly show that 7 – 27% of newly established OTF herds were antibody positive over the duration of observation.

**Figure 4:**
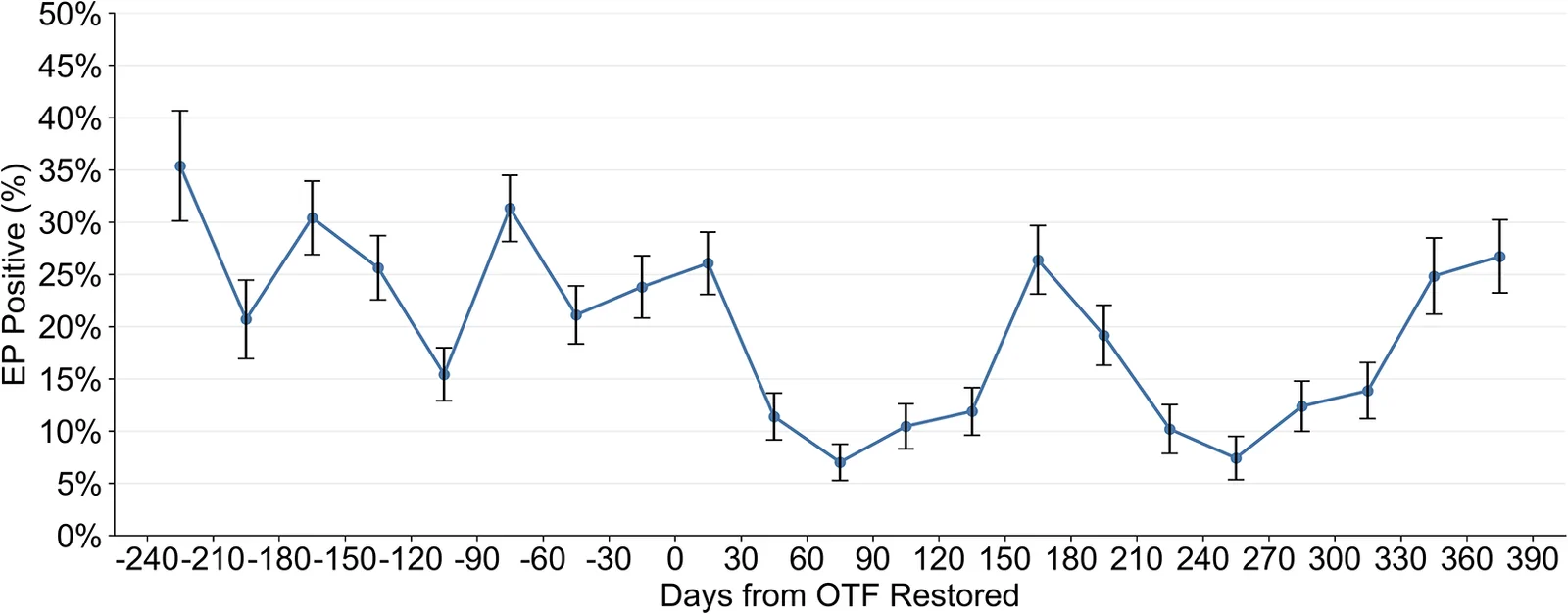
Percentage EP positivity against days before and after lifting of restriction in newly defined OTF herds. The data for percentages are expressed as mean +/- standard error. The X-axis shows days from OTF restoration. The Y-axes show percentage of EP positive herds. The number of herds sampled ranged between 167 and 226 across the observed period.

### Effect of monthly testing on EP positivity

Seventy-three herds were recruited separately and provided a monthly bulk milk sample for analysis. Apart from three herds which were in the EA, all were located in the HRA. The data are shown in **Table 7**. The results show that in OTF herds, 51% were EP positive when tested serially six times compared to 35% when tested quarterly. In restricted herds, 64% were EP positive when tested serially six times compared to 45% when tested quarterly. The data thus shows that, regardless of whether a herd was under restriction or OTF, there was a significantly higher proportion of EP positivity detected by monthly rather than quarterly sampling.

**Table 7:** EP probability with each round of monthly testing in restricted and OTF herds.

Round comparison ^1^	OTF^2^ herds (n = 51)		Restricted herds (n = 22)	
	Increase in probability of obtaining at least one positive EP test result	P value	Increase in probability of obtaining at least one positive EP test result	P value
1 > 2	0.08	**P = 0.01**	0.14	**P < 0.01**
2 > 3	0.09	**P < 0.01**	0.09	**P < 0.01**
3 > 4	0.08	**P < 0.01**	0.06	**P < 0.01**
4 > 5	0.07	**P = 0.02**	0.05	**P = 0.05**
5 > 6	0.06	P = 0.22	0.05	P = 0.31

1 Comparison between successive BTM samplings. P values < 0.05 were regarded as being statistically significant and shown in bold.

2 OTF, officially tuberculosis free.

**Figure 5** shows the effect of increasing the frequency of sampling on EP positivity in both bTB restricted and OTF herds. **Figure 5A** shows progressive increases in the EP positivity with the number of times the herd is sampled, ranging from 25% (Sample 1) to 64% (sample 6) in restricted herds. **Figure 5B** shows the same effect in OTF herds. In both cases, the overall effect was statistically significant (P<0.05). Progressively increasing the number of samplings by one was statistically significant (P<0.001) from 1 to 5 samplings but became insignificant when the frequency increased from 5 to 6 samples (P>0.05). Statistical analysis showed that the number of EP positive samples in restricted herds was marginally higher than that in OTF herds (P = 0.05). The increase in EP probability at each sampling is shown in **Table 8**. The increase in EP positivity at each round of testing ranged from 6% to 9% in OTF herds and from 5% to 14% in restricted herds. The increase in EP positivity was statistically significant up to 5 rounds of testing in both OTF and restricted herds.

**Figure 5:**
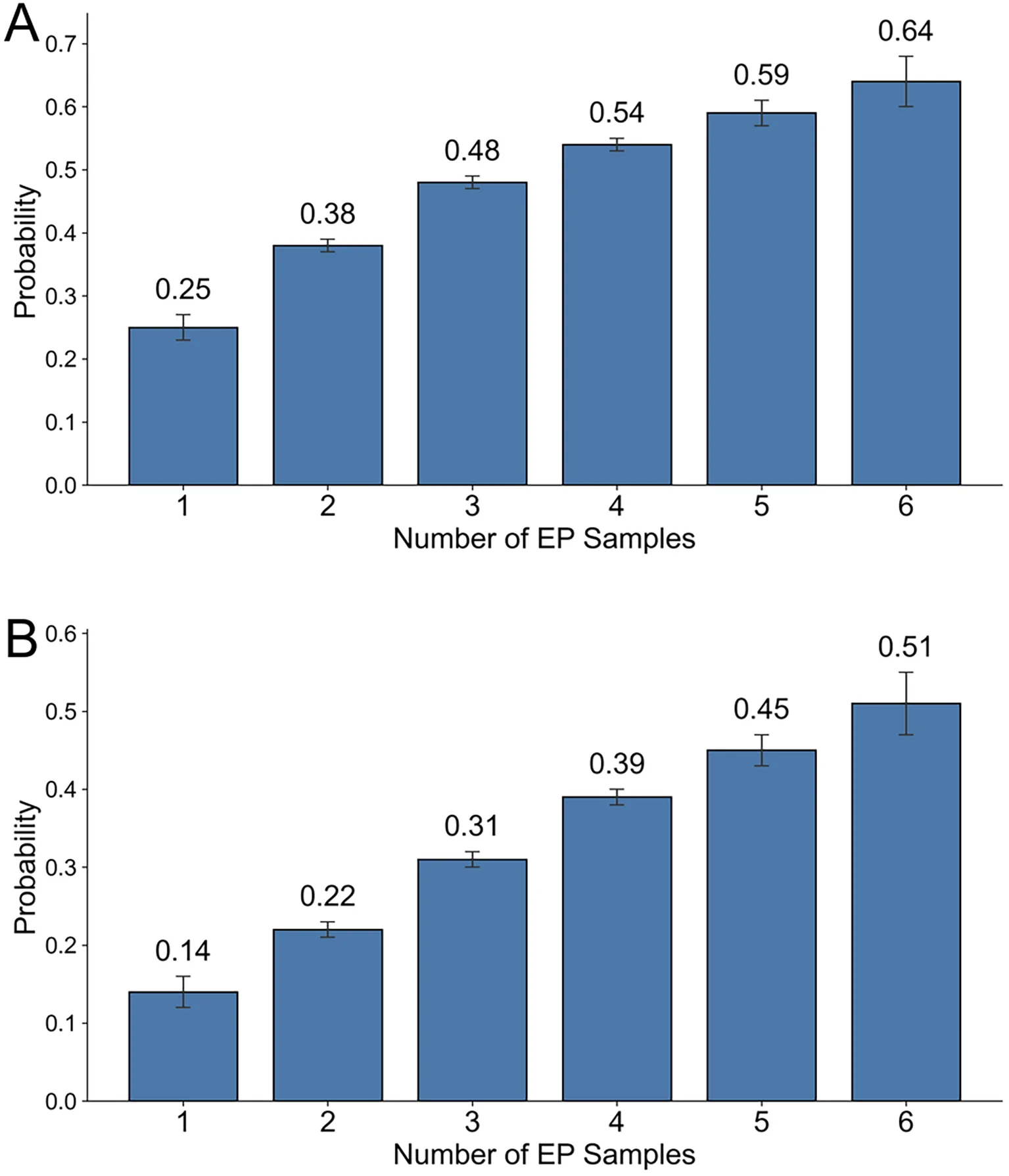
Probability of EP positivity in herds under restriction **(A)** or herds that were OTF **(B)** during trial tested monthly for 6 months. The bars represent mean +/- standard error. The Y axis shows probability of EP positivity. The X axis shows the number of times the herds were sampled. The EP probability is shown attached to each bar. Values for 1–5 samples represent the average of all possible combinations respectively. The data were analysed by univariable logistic regression with an F-test to assess statistical significance using the average of all possible combinations of 1, 2, 3, 4 and 5 tests.

**Table 8:** The number and percentage of herds found EP positive by their bTB status when sampled every month.

bTB restriction status^1^	Monthly testing (1–6 months)	Quarterly testing (1, 3, 6 months)
	*Number EP positive/tested (%)*	*Number EP positive/tested (%)*
Negative	26/51 (51%)	18/51 (35%)
Positive	14/22 (64%)	10/22 (45%)
Total	40/73 (54.8%)	28/73 (38.4%)

1 Herds were either under statutory movement restrictions (Positive) or were officially tuberculosis free (Negative).

## Discussion

### Minimising the EP false positive rate

To ensure confidence in the test results in this study, we attempted to minimise the false positive rate in GB samples. To achieve this, we used asymptotic regression analysis (15) to develop more stringent criteria for selecting the bTB free herds and, in addition, we applied a minor alteration to two of the antigen cut-offs. This achieved our target for our FPR to be below 1%.

When analysed by distance the sample OTF herd was from a herd under bTB restrictions, the estimated FPR at the herd level dropped progressively from 4.5% at 0 km to 0.8% as the distance from a breakdown herd increased and reached an asymptotic maximum around 16 km. This result contrasts with the analyses of CCT test specificity shown by Goodchild and colleagues (15) where the FPR estimates dropped from around 1.0% to 0.002% as the distance increased, reaching asymptotic maximum around 8 km. The reason the FPR estimates at the zero km distance point were 4.5% for EP against 1.0% for the CCT test is almost certainly due to the EP estimates being determined at the herd level, whereas for the CCT test, it was determined at the individual animal level. This may also explain why the distance from an infected herd for maximal apparent EP specificity was found to be twice that of the CCT.

Goodchild and colleagues (15) stated that in an average sized herd in their study (n=45), which utilised data collected from all routine herd tests conducted in GB between 2002 and 2008, the CCT test herd level FPR would be between 0.8% and 0.5% which is similar to that found for the EP in this study. They also concluded that tests of larger herds were likely to suffer from a higher FPR. For example, if a herd with 250 animals were tested, the expected herd level FPR would be around 3.5%. In the present study, the average milking herd size was estimated to be 241 using number of animals calved as a proxy, suggesting that the CCT FPR would be close to the 3.5% figure quoted for 250 animals above. This suggests that in this study, the EP demonstrated a higher apparent herd-level specificity than that estimated for the CCT test.

Samples taken in Round 1 gave a higher FPR estimate than those taken in Rounds 2 and 3. The reasons for this are not known. The proportion of samples taken in the anamnestic period were higher in Round 1 compared to Rounds 2 and 3 and these gave higher EP positivity results. However, the numbers of bTB free herds which were sampled in the anamnestic period were very low and the confidence intervals of the FPR are very wide and therefore caution should be applied when interpreting these results. Previous work using serum samples showed no increase in FPR when samples were taken during the anamnestic period in bTB-free animals (37, 39). The latter is consistent with the lack of antibody positivity in experimental bTB-free animals repeatedly injected with tuberculin (28, 32–34, 47–49).

Because this was a survey rather than a validation study, we did not conduct a detailed assessment of the EP’s analytical specificity. This had already been evaluated in our previous work (37–39), which found that non-tuberculous mycobacteria (NTM) had no significant effect on EP specificity using individual-animal serum or milk, or in bulk milk. Given MAP is the most common NTM in GB, with 68% of dairy herds thought to be infected (50), this data would also suggest that there is no significant cross reaction between MAP infection and the EP.

### Antibody positivity in all herds

Analysis in all herds participating in the study showed that the HRA, EA and LRA contributed 74%, 17.5% and 8.5% of EP positivity respectively. Although the datasets are not completely analogous, the APHA analysis of breakdowns in England in 2023 showed that the HRA, EA and LRA contributed 76%, 18%, and 6% respectively (46) suggesting that the amalgamation of the risk areas across England, Scotland and Wales in this study had produced comparable outcomes. It should be noted that over 99% of herds in this study were sampled when CCT reactors were not known to be present. A very small number of herds had BTM samples taken on the day of reading the CCT test when a reactor was disclosed. However, it was not known whether any of these reactors contributed milk to the bulk tank.

### Antibody positivity in OTF herds

OTF status implies that a herd is free of bTB. However, evidence of undisclosed infection in OTF herds has been accruing over the last few years (8). For example, epidemiological modelling showed that 24% or 50% of recurrent breakdowns in the UK could be attributed to infection being missed by current testing regimes depending on the model used (51) though this model was produced before adoption of an enhanced frequency of CCT testing and increased use of IFNg tests in England so these percentages may not be representative of the current situation in England. Visible lesions with *M. bovis* culture positive confirmation have been detected at slaughter in culled OTF animals (52, 53). Similarly, antibodies have been detected in CCT negative, *M. bovis* culture positive animals with visible lesions (37). It is estimated that about 20% of infected animals are not detected by routine CCT testing in GB and Northern Ireland (54). In this study, we found 19.4% of OTF herds were EP positive in BTM samples, a result which is consistent with the above estimates. If the bTB-free herds were removed from the OTF cohort, a slightly higher EP positivity rate (21.6%) was observed. It should also be noted that the BTM samples from OTF herds were mainly taken during the non-anamnestic period, and it is possible that even higher rates of EP positivity would have been found if sampling was solely anamnestic. The EP positivity observed showed the HRA as having the highest percentage level of positive herds, followed by the EA and then the LRA, and the relative risk of being EP positive increased from 7.4 to 31.0 to 39.8 in LRA, EA and HRA respectively compared to bTB-free herds. These results are consistent with known risks associated with the bTB risk area assignments and suggest the presence of undisclosed infection in these herds.

The EP positivity observed in the three rounds of testing in OTF herds showed that Round 1 produced the most positives, followed by Round 3 and then Round 2. Differences in the proportion of samples taken during the anamnestic window following a skin test could contribute to this pattern. However, other factors may have played a role. For example, winter housing brings cattle closer together, facilitating bTB transmission within herds, and *M. bovis* survival is greatest in cool, moist, shaded environments (55–59). Additionally, based on AHDB survey data, approximately 10% of herds in the study population are likely to have used a Spring Block Calving production system which is associated with lower average milk yields per cow at the start and end of lactation which could influence the EP positivity rates (60). Analysis of potential confounding factors for EP positivity including milk yields will be reported in a separate publication. A failure to detect infected OTF herds is likely to contribute to the perpetuation and spread of bTB within and between herds. OTF herds are not under movement restrictions, so animals can be traded freely to other OTF herds though individual animals must undergo a pre-movement test if the movement occurs more than 60 days after a whole herd tuberculin surveillance test (61). Detection of these infected herds by EP testing in conjunction with a requirement for animals from EP positive herds to be pre-movement tested with WOAH approved more sensitive tests would reduce this risk. Analysis of risk of the subsequent loss of OTF status in herds that imported animals from EP positive OTF herds will be reported in a separate publication.

Given that it only requires for one animal within an infected herd to be determined as a reactor for the herd to be placed under restriction, these results must place further scrutiny on the sensitivity of the CCT test. OTF herds are deemed to be bTB free through statutory testing. This implies that the EP positivity found in these herds could be due to detection of infected animals missed by the statutory tests. This conclusion is supported by the observation that 90% of these herds had undergone a whole herd TT within the study period. In GB, statutory testing varies between the HRA (every 6 months), EA (either every 6 months or annually), and LRA (every 4 years or abattoir surveillance only in the case of some Scottish herds), and the proportion of herds diagnosed with bTB in each area reflects this. The EP positivity rates observed during the study showed a rank order of HRA> EA>LRA and were consistent with the statutory risk area ranking.

Recent individual animal sensitivity estimates for the CCT test range from 40% to 100% (15) and 61.3% for herd level sensitivity (18) indicating significant scope for diagnostic failure using the CCT test for surveillance and pre- and post-movement testing. Analysis of false negative results from the CCT test in Northern Ireland showed that the test missed 320 *M. bovis* culture positive animals out of 1,891 CCT test negative animals (16.9%) (16). Though the overall data suggests that, in many cases, EP positive OTF herds that previously had periods of bTB restriction are likely to be a result of a failure of current surveillance to remove all infected animals from the herd, it should be stated that the potential for such herds to have become re-infected exists. This could be due to the purchase of replacement animals, contact with neighbouring infected cattle, transfer of infection from local wildlife reservoirs, or environmental sources of infection. These factors could also explain the observed outcomes.

These results indicate that the CCT test on its own lacks the diagnostic power even at the herd level to detect and control bTB efficiently. This is a concern given the significant costs associated with its use. APHA data showed that in 2023, 5.2 million TB skin tests and 72,008 herd tests were carried out in bovine animals. Defra has estimated that the associated cost of each skin test event was circa £715 in 2023 - 2024, a skin test event being defined as including the whole herd (usually all the animals of at least 42 days of age), a proportion of a herd, or a few individual animals in a herd (57). This would suggest that the 72,008 herd tests carried out in 2003 (62) cost the UK Government a minimum of approximately £51.5 million pounds. Furthermore, these costs do not cover the private costs incurred by the farmer for submitting animals for testing.

### Antibody positivity in restricted herds

In herds that were under restriction throughout the study period the EP positivity was 40.4% overall, with the relative risk of being EP positive over 50 times that of bTB-free herds. In restricted herds, a short interval test (SIT) is applied at a minimum interval of every 60 days and therefore the number of anamnestic antibody responses is likely to be increased accordingly. Herds that regained OTF status during the study showed high EP positivity while restricted, but lower and more variable levels of positivity after restoration of OTF status. This result is likely to be due in part to OTF herds being subjected to fewer CCT tests, as well as to natural host-pathogen interactions. However, the EP positivity rate ranged between 7 and 27% in these OTF herds over the course of the study, suggesting the presence of residual infection despite removal of the CCT test reactors. This residual infection has the clear potential to contribute to the spread of infection within and between herds (9), though it is also possible that infection is being controlled and spread of infection is limited. Such antibody positivity provides a partial explanation for the persistence of the disease in the national herd. Testing restricted herds with the EP test thus provides an easy means of determining whether potentially infected animals remain in the herds after removal of the CCT test reactors. Individual animal testing could be applied to EP positive herds to identify the potentially infected animals contributing antibody positive milk to the BTM sample.

### Monthly testing

The results discussed above relate to those obtained following three rounds of quarterly BTM testing. We also examined whether monthly testing would provide a further enhancement of the rate of detection. The data showed that EP positivity rates increased using serial testing. The results in this study for both restricted and OTF herds show that there is a significant increased risk that a herd will demonstrate an EP positive result when the frequency of testing is increased from three monthly intervals to monthly intervals over a period of six months. This results in an approximate 8 - 10% increase in the number of herds testing positive for every extra month of surveillance at least up to month 5. Further work is required to determine how long this effect continues for. The restricted herds in this cohort would have been subjected to 1–3 SITs during the sampling period, resulting in anamnestic boosting of antibody responses. Outside the LRA, the OTF herds would receive routine tuberculin tests every 6 months and so some anamnestic boosting may have been present which could have increased antibody levels. The slightly higher probability values observed in the restricted herds compared to the OTF herds could therefore be due to higher rates of anamnestic boosting.

Serial sampling would potentially result in accumulation of false positive results if no action was taken. However, assuming that the FPR was the same in these herds as that observed in **Table 5** for herds free of bTB (0.8%), then the expected number of false positive results in these restricted herds and OTF herds would be 0.02/22 herds and 0.04/51 herds respectively. In larger datasets, the FPR of 8/1057 would apply. The results in this small cohort clearly show that adoption of monthly testing would result in significantly increased detection of potentially infected herds. It should be noted though, that except for three herds that were in the EA, all the herds tested monthly were in the HRA and therefore this dataset is clearly not representative of the whole GB dairy industry. The data suggest, however, that the percentage of herds infected in GB shown in this study is very likely to be an underestimation of the true level of endemic infection.

## Conclusions

The EP test demonstrated a very high herd-level specificity. We have demonstrated that approximately 19% of herds with OTF status and 40% of herds under bTB restrictions respectively were antibody positive by the EP test in one or more BTM samples. The detection rate of potentially infected herds by the EP test, both OTF and under bTB restrictions at the time of BTM sampling, significantly increased with the frequency of sampling. The results overall indicate that current surveillance for bTB in GB, based primarily on statutory testing with the CCT test, misses statistically significant numbers of herds that are highly likely to be infected with *M. bovis*. Screening for antibodies to *M. bovis* using the Enferplex BTM milk test would be a convenient, cost-effective way to supplement the existing CCT herd testing programme and routine post-mortem surveillance of cattle carcasses in abattoirs to improve detection of bTB in dairy herds in GB.

## Data Availability

The data analysed in this study are restricted to authorized users due to legal restrictions placed upon SRUC by APHA. Requests to access the datasets should be directed to Professor Gordon D. Harkiss. info@mvdiagnostics.co.uk.
